# Identifying families’ shared disease experiences through a qualitative analysis of online twin-to-twin transfusion syndrome stories

**DOI:** 10.1186/s12884-016-0952-6

**Published:** 2016-07-15

**Authors:** Rebecca Fischbein, James Meeker, Julia R. Saling, Michelle Chyatte, Lauren Nicholas

**Affiliations:** Department of Health Policy & Management, College of Public Health, Kent State University, 800 Hilltop Drive, Moulton Hall, P.O. Box 5190, Kent, OH 44242-0001 USA; Northeast Ohio Medical University, 4209 St. Rt. 44, P.O. Box 95, Rootstown, OH 44272-0095 USA; D’Youville College, 320 Porter Avenue, Buffalo, NY 14201 USA

**Keywords:** Twin-to-twin transfusion syndrome (TTTS), Qualitative, Emotions, Psychosocial, Lived experience

## Abstract

**Background:**

Twin-to-twin transfusion syndrome (TTTS) affects 10–20 % of monochorionic diamniotic (MCDA) births and accounts for 50 % of fetal loss in MCDA pregnancies. This exploratory qualitative study identified shared experiences, including potential emotional and psychosocial impacts, of this serious disease.

**Methods:**

Forty-five publicly accessible, online stories posted by families who experienced TTTS were analyzed using grounded theory.

**Results:**

Shared TTTS experiences included a common trajectory: early pregnancy experiences, diagnostic experiences, making decisions, interventions and variable outcomes. Families vacillated between emotional highs such as joy, excitement and relief, and lows including depression, anxiety, anger and grief.

**Conclusions:**

TTTS disease experience can be considered an “emotional roller coaster” exacerbated by TTTS’s unpredictable and quickly changing nature with the potential for emotional and psychosocial effects. Increased TTTS awareness and research about its corresponding impacts can ensure appropriate patient and family support at all phases of the TTTS experience.

## Background

Twin-to-twin transfusion syndrome (TTTS) is a rare but serious fetal disorder occurring with a prevalence between 1 and 3 in 10,000 births [1] and 10–20 % of monochorionic diamniotic (MCDA) births [2]. Approximately 17 % of twin fetal death is due to TTTS and 50 % of fetal loss in MCDA pregnancies is attributable to the syndrome [1].

TTTS results from a mismatch in angioarchitecture, providing the recipient twin with excess blood volume while blood supply to the donor twin is diminished [3]. TTTS is divided into five stages of increasing severity: Stage I presents as discordance in amniotic fluid in an MCDA pregnancy with progression to Stage V defined as death of one or both twins [4]. Approximately 15 % of Stage I pregnancies will proceed to a higher TTTS stage [4]. The progression of TTTS is highly variable and survival depends upon stage and gestational age at onset of the syndrome. Without treatment, prognosis for pregnancies in Stage III or higher is grim with a fetal loss rate of 70–100 % [4]. Treatment options include amnioreduction, fetoscopic selective laser ablation surgery, intervening membrane septostomy and selective reduction. It is recommended that chorionicity be identified in all twin pregnancies between weeks 10 and 13, with MCDA identified gestations referred to a maternal fetal medicine (MFM) specialist by primary care physicians so that bi-weekly ultrasounds and consultations can begin at week 16 [4].

Little information exists concerning the parental disease experience among families who experience TTTS pregnancies. Research by Nicholas (2014) represented the first time the disease experience was examined for TTTS families, finding that physician characteristics and sociodemographic factors were related to TTTS survival outcomes [5]. Prior work by Edwards et al. (2007) demonstrated a link between negative emotional and stress levels associated with fetoscopic selective laser ablation surgery treatment for TTTS pregnancies [6].

### Research question

Given the potential impact that the TTTS experience may have on families, it is important to determine if there are shared disease experiences among these families. Uncovering the common experiences of TTTS may raise awareness of possible comorbid mental health manifestations, assist health care professionals when treating families with TTTS as well as provide newly diagnosed patients with greater information and sources of support. In addition, understanding shared TTTS disease experiences can serve as a foundation for future research. Therefore, the following research question will be addressed through this study:

1. What, if any, are the shared disease experiences among families who experience TTTS?

## Methods

### Data collection

Data for this study were obtained from family members’ stories about TTTS posted on the Fetal Health Foundation website (http://www.fetalhealthfoundation.org). Stories were included in the analysis if written by a parent or family member experiencing a TTTS pregnancy. Forty-six stories involving TTTS pregnancies were initially identified. However, one story failed to meet the authorship requirements of this study. Therefore, a total of 45 stories were included for analysis. Once identified as data, stories were stored and analyzed using Qualrus (Idea Works, Inc. 2002) software. This research protocol was approved by the Institutional Review Boards at Kent State University and Northeast Ohio Medical University as meeting requirements for exempt research.

### Analyses

This qualitative study utilized a grounded theory research design. Grounded theory is used to construct theoretical models from the analysis of qualitative data such as interviews or personal narratives [7]. These new theories are therefore “grounded” in the social data collected [8]. Use of the grounded theory technique allowed us to explain how parents and family members experienced the TTTS pregnancy. Furthermore, analysis of the data through grounded theory permitted us to construct a new theoretical model of the experience of a TTTS pregnancy.

Grounded theory methods include the analysis of social narratives and the development of codes, categories and themes based on their description and content [9]. Accordingly, data were analyzed using a series of coding stages: (1) open coding, (2) axial coding, and (3) selective coding [10]. First, data were open coded, in which the family members’ experiences were organized based on their meaning into theme-based categories. Second, in the axial coding phase, categories were compared to one another to examine inter-relationships. Since qualitative research is an open-ended process, new categories were developed during data collection as new information became available; in this fashion grounded theory allowed the investigators to develop new insights that were reactive to the data being analyzed [11]. New categories were defined into subcategories, allowing the analysis to evolve in response to the subject material as it became more complex. [12, 13] Lastly, through selective coding, a primary theory of the experience of TTTS pregnancy was developed based on the relationships of data categories and meanings [10]. Since this was a qualitative analysis, ample use of direct ‘in vivo’ quotes from the stories are used as indicators of the particular themes and experiences uncovered from the data to support the new theories being generated [14].

Throughout the study the first and second authors met to analyze data, provide feedback, develop categories, and challenge each other’s assumptions and subjective experiences. To reduce bias, both authors coded independently and worked together to reach consensus on the analyses [15]. This method of collaborative research enhanced the validity and rigor of the study.

## Results

Included in the study were 45 stories posted on the Fetal Health Foundation website by families who were diagnosed with TTTS. Table 1 provides information about the stories, pregnancies and outcomes. The majority of stories were reported by mothers (80.0 %). Forty-three (95.6 %) stories told of identical twin pregnancies while two of the stories included triplets, both with identical twins and a fraternal triplet. The dates of the stories ranged from 1998 to 2011 with the median story occurring in 2006. All but two posts (95.6 %) indicated that TTTS diagnosis was made prior to birth. Of the cases diagnosed prior to birth, on average, the TTTS diagnosis was made during the 18th week of pregnancy (average 18.6 weeks, range 13 to 26 weeks). Treatment methods described by families varied, with the majority reporting fetoscopic selective laser ablation surgery (*n* = 26, 57.8 %) followed by amnioreduction not occurring during fetoscopic selective laser ablation surgery (*n* = 12, 26.7 %). The average week of birth for families with at least one live birth was 30.9 weeks, while 24.0 weeks was the average week of birth for families with no live births. Of 92 total fetuses, 71 were live births (77.2 %) and 66 (71.7 %) were reported surviving. Approximately 76 % (*n* = 31) of families with a live birth spent time in the Neonatal Intensive Care Unit (NICU) and of those, 38.7 % (*n* = 12) experienced serious complications while in the NICU.

**Table 1:** Summary Content of TTTS Stories, Pregnancies and Outcomes

		N	%
Author of story			
Mother		36	80.0
Father		7	15.6
Maternal Grandmother		2	4.4
Pregnancy Type			
Twins		43	95.6
Triplets		2	4.4
Year of pregnancy (median, range)		2006 (1998–2011)	
Diagnosed after birth		2	4.4
Weeks at Diagnosis (mean, sd)		18.6 (3.2)	
Treatments			
Nutritional Supplementation		3	6.7
Amnioreduction^a^		12	26.7
Surgical			
Cerclage		1	2.2
Septostomy		1	2.2
Fetoscopic selective laser ablation surgery		26	57.8
Selective Reduction		2	4.4
Immediate delivery		3	6.7
Wait and see until viability		1	2.2
Weeks at birth (mean, sd)			
Two plus live births		31 (3.6)	
At least one live birth		30.9 (3.9)	
No live births		24.0 (5.7)	
Outcomes			
Live births		71	77.2
Stillborn		21	22.8
Survivors		66	71.7
Outcomes by family^b^			
Two survivors		30	66.7
One survivor		11	24.4
No survivor		4	8.9
NICU stay by family (at least 1 live birth)			
Yes		31	75.6
No		6	14.6
Missing		4	9.8
Serious complication while in NICU by family			
Yes		12	38.7
No		19	61.3

^a^Does not include amnioreductions that occurred during fetoscopic selective laser ablation surgery

^b^2 triplet families’ outcomes: 1) one fraternal survivor, 2) a TTTS survivor and fraternal survivor

**Fig. 1:**
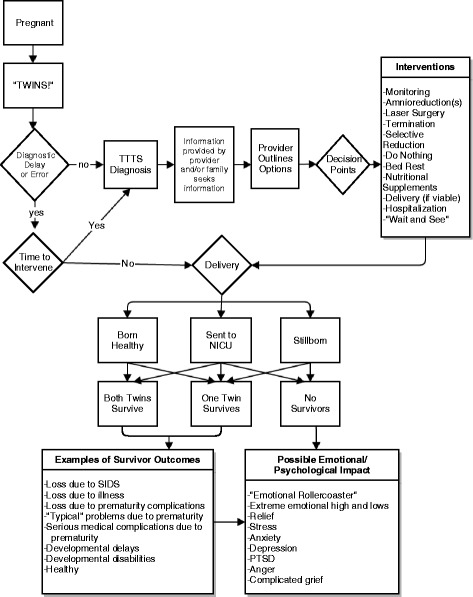
Visual Depiction of Shared TTTS Disease Experiences

Although the specific course and outcomes associated with the disease differed, shared TTTS experiences described by families followed common trajectories (see Fig. 1). Likewise, five chronological themes emerged from the stories: early pregnancy experiences, diagnostic experiences, making decisions, interventions and outcomes. Several subthemes emerged within most themes.

### Early pregnancy experiences

The majority of families (82.2 %) relayed early pregnancy experiences prior to TTTS complications or diagnosis. Subthemes dealt primarily with emotions surrounding the discovery of a twin pregnancy, the mother’s or bodily intuition that there was a problem with the pregnancy, and, as experienced by a few families, the lack of time to act to save the lives of their unborn children.

#### It’s Twins!

Parents described a flood of emotions including excitement (48.8 %), shock (37.7 %), fear (11.1 %), feeling overwhelmed (8.8 %) and anxiety (6.6 %) upon discovering their twin pregnancy.

#### Mother’s/Bodily intuition

The emotional high associated with the discovery of identical twins abruptly ended with the onset of complications or signs of TTTS. Thirteen families (28.8 %) recounted experiencing a symptom that was an indicator of TTTS, such as pain, gaining weight quickly or shortness of breath, that was missed by their health care provider. Eight mothers described having had a sense of bodily or mothers’ intuition that something was not right. One mother relayed how her concerns about her increasing size and sense that something was wrong were downplayed by medical staff. Another family explained that mother’s intuition warned them something wasn’t right and led the mother to demand an ultrasound.

#### No time to act

Two families revealed that although signs of TTTS had been observed, the disease progressed too quickly for intervention and both sets of twins passed away. Two additional families shared there was no time for intervention and their babies passed away due to undiagnosed TTTS. TTTS was only mentioned to the families after the pregnancy ended. Cases in which families underwent an entire TTTS pregnancy with no knowledge of the condition and subsequent fetal death were limited to the early 2000’s, specifically 2003 and 2004.

### Diagnostic experiences

Emotional reactions differed among the 43 families who received a diagnosis during the pregnancy. Nine families (20.9 %) relayed being shocked and stunned by the diagnosis: “Everything in our world came to a screeching halt”. Seven families (16.3 %) recounted feeling hopeless after receiving the news. These families described feeling as though there was no chance for their babies’ survival. Two families’ stories shared the confusion and difficulty understanding the medical information that followed the diagnosis: “It was a foreign language; I had no idea what he was talking about. It was only when we were taken to a meeting room with Kleenex on the table, did the severity begin to set in.” Another family revealed: “All I could do was hold my husbands [sic] hand and turn to him and cry. I surmised all the courage I could muster and tried to understand the flow of medical options that came next.” The amount and type of information shared by providers during the diagnostic process differed. Seven families (16.3 %) stated they were told by a healthcare provider their babies would die. Further, seven families (16.3 %) were initially only given the name of the syndrome or a referral to a specialist with no additional information.

#### Seeking information

Upon being diagnosed many parents sought out more information. Almost half of all families (47 %) conducted their own research to learn more about the disorder. The main sources of information included online searches (*n* = 14, 32.6 %), contacting foundations (*n* = 6, 14.0 %) and independently seeking out the advice and help of specialists (*n* = 8, 18.6 %).

### Making decisions

Parents were provided a series of options for the pregnancy. Table 1 describes the treatments and choices the families made. The options for families depended upon the stage of the syndrome, specifics of each pregnancy and wishes of the family. The choices were clear for some parents and less obvious for others. One family described feeling the only option for them was amnioreduction: “The choices we had were to terminate the pregnancy, amnio-reductions, surgery or let nature take its course. I was not a candidate for the laser surgery since I had bleeding. I was not going to terminate the pregnancy. I wanted to do everything I could to save my babies so the amnio-reductions were the only way to go for us.” Another family explained surgery was recommended but conversations with an independently sought specialist caused them to question their first doctors’ suggestions:

Our doctors wanted us to make a decision. Through our conversations with [specialist] and reading the literature, we gained the knowledge to help us feel as though we had some control… We felt torn as we were being persuaded towards surgery…We went home praying for guidance.

Some parents had to make incredibly difficult decisions. The hope associated with possible interventions quickly turned to despair when those interventions were not an option:

I contacted a doctor in Los Angeles for a second opinion. He admitted that laser surgery would most likely result in the death of both boys. My ray of hope turned into devastation. My husband and I discussed and prayed for help in this difficult decision, a decision that no parent should ever have to make. I questioned everything…we finally decided that we couldn’t lose both boys and that if our only option was to save one or lose both we would have to save the one…I was able to see Javier^1^ on the sonogram before the procedure. He wasn’t moving very much, but I saw his little heart beating. I begged for his forgiveness at what we were about to do.

### Interventions

Once families had made a decision about how to proceed with the pregnancy, the treatment options chosen by the families included: fetoscopic selective laser ablation surgery, amnioreduction, nutritional supplementation, immediate delivery, selective reduction, “wait and see” until viability, cerclage and septostomy. The following passages illustrate how dramatically different outcomes could be after surgery and the speed and degree to which emotions changed. One family revealed their fear and subsequent excitement upon discovery of the heartbeat of both twins:

Before the ultrasound I was very, very scared. And everything that I had feared came back ten fold. If this surgery didn’t work, what else did we have. The ultrasound was underway and right away there was our Gavin’s heartbeat, I started to cry. Then with the grace of God our little Tyler’s heart was beating away and God Bless his little bladder was visible!!

Another family shared how excitement and optimism after surgery turned to devastation upon learning one of their twins had not survived:

The day started out great. I felt strong movements and we had a good feeling about the fetal echocardiogram that we were scheduled to have later that morning. We went over to Children’s Hospital and were excited to find out how both of our boys where doing. Expecting to hear from the doctor that both of the boys where [sic] going to be keeping me uncomfortable for the next 13 weeks. Then our world came to a sudden and frightening halt. The words “I'm sorry, your baby’s heart isn’t beating” will be forever etched in my brain. We were completely blown away and devastated. And still expected to lie still while the cardiologist checked out our surviving twin.

During and after treatment, families often faced long waits for viability and delivery. These waits could be uneventful or full of more complications and stressors. For example, one family reported that after surgery the syndrome reversed and described the subsequent feelings of hopelessness:

The TTTS has reversed. Our original donor Tyler was now our recipient and Gavin being the original recipient was now the donor. I could not believe it. We were past the point for another surgery and we were not at the point for the babies to be viable. What were we supposed to do now. At this point I was so defeated I didn’t know what to do.

Although intervention offered some hope for a positive outcome for these families, the experience was fraught with highs and lows, as there was no way to predict how their pregnancy journey would unfold during or after intervention.

Four families conveyed the time between diagnosis, decision-making and intervention using phrases such as “a race against time”, illustrating the sense of urgency experienced by the families during these stages. In the midst of this rush, many families needed to travel out of town to go to a different hospital where diagnostic and treatment options were available (*n* = 18, 41.9 %). One mother explained the stress and urgency she felt while waiting to see whether insurance would cover the cost of treatment:

Wednesday was the most difficult day of my life, waiting for a call to indicate that my insurance company had approved the consultation and surgery. Finally the call came late Wednesday afternoon, and we were set for our consultation the next morning. I don’t think I slept much that night. Knowing how quickly TTTS can progress, I wondered if the babies were even still alive?

### Variable outcomes

All families shared outcomes of their TTTS pregnancy, which included: two survivors (66.7 %), one survivor (24.4 %) and no survivors (8.9 %). One challenge faced by the majority of parents, regardless of outcome, was time in the NICU with approximately 76 % of all families having at least one infant stay in the NICU. Nearly 39 % of those families described serious NICU complications such as intraventricular hemorrhage (IVH), kidney malfunction, congenital heart defects and placement of a feeding tube. One mother recounted the emotional impact of discovering her son had necrotizing entercolitis (NEC):

When I walked into the NICU, I immediately saw that his stomach was blown up like a balloon. He just lay there, motionless. I was shown X-rays of little gas bubbles in his intestine. Someone said something about NEC--Necrotizing Encroloitis [sic]. Yet another term I could place hatred on.

#### Two survivors

Families shared stories of two live births ranging from healthy newborns with good birth weights to stories of infants experiencing serious medical complications. Approximately 67 % of stories concluded with two survivors. One maternal grandmother with two survivors empathized with others experiencing TTTS:

“I know the feelings of dread, panic, agony of the waiting period. The just NOT knowing from day to day, month to month how things will turn out.”

#### One survivor

Almost 25 % of families had a single survivor. The majority of families lost their twin before birth, however, five infants were lost in the days or months following birth. The following passage relays the experiences of one family whose infant faced severe NICU complications and subsequent grim prognosis:

Everything seemed to be going great until they told us that he had developed a grade 4 brain hemorrhage. They said that with that type of hemorrhage, there was no chance at him surviving. We were given a decision to make, pull the life support or continue to keep it going. We were asked to go home and sleep on the decision before we made it. We went back on Friday morning. We were prepared to make our decision and pull all life support.

Parents with one surviving baby and one stillborn shared the pain of learning one twin had passed:

When they did the ultrasound the tech looked at me and said Beth, Grace has no heartbeat. All I could say was NO! I was in shock. No emotion. No tears. Then she went to get the Doctor. As soon as she left it hit me. I lost my little girl Grace. I screamed and cried. I could not believe all that we had done and everything looked good the day before.

The same mother revealed the complex mix of emotions surrounding having one survivor:

Then they delivered Grace (stillborn). In recovery we were able to spend time with Grace. She was beautiful… She had such long lashes and such tiny hands. I cried as I held her… We spent about 2–3 h with her not knowing how our other baby girl was doing in NICU. I didn’t want to let go. I was devastated. I had such mixed emotions, I wasn’t sure if I was ready to see her. Finally I went to see her, OH MY she was so tiny.

Two other families described that seeing their surviving twin always caused them think of the other, lost twin. One mother reported: “I look at Juan and can’t help but think of Javier by his side. They will forever be brothers!”. Likewise, Grace’s mother explained: “She is truly a miracle and as I see her I know Grace would look just like her. I still have some bad days but they are not horrible. Grace is always in my thoughts. There is not 1 day that goes by that I don’t think of her.”

#### No survivors

Almost 9 % of families suffered the loss of both twins. Families with no survivors recounted the emotional impact of losing both twins including an incredible amount of grief, the loss of an imagined future, feelings of emptiness and anger. One maternal grandmother expressed the pain of watching her daughter give birth to her twins lost to undiagnosed TTTS: “My daughters [sic] next 2 days were filled with pain and agony of having to go through labor to deliver her two dead babies. I have never witnessed such a heart wrenching experience in my life…” In the following passage, a mother who lost both twins described the immediate and longer-term impact of the loss:

I felt like a zombie, walking around my house. I would feel my stomach, it would be so flat and I would just break down. My husband comforted me in every way that he could. I knew though that he was grieving too. I tried to resume life as normally possible, but it was impossible. I got a job, and my husband went back to work. Then things spiraled down. We were late for work every day. I was on the verge of getting fired. I always asked myself WHY?? WHY ME?? I stopped cleaning, taking care of myself, working out, cooking dinner. We were sad all the time, just feeling sorry for ourselves and pissed off at whoever didn’t feel sorry for us as well. We lost friends and didn’t make new ones.

One mother shared the grief that accompanied the loss of her twins and how she coped:

I would not have either of them to bring home. All I would have to bring home was a terrible feeling of emptiness inside. I still have a lot of pain, anger and sadness inside me. My husband and I bought our own grave sites and the girls are buried at the foot of my grave. I visit them frequently, it gives me some peace.

## Discussion

This research is the first to examine shared disease experiences of a TTTS pregnancy. When examining the pregnancy stories provided by 45 families who experienced TTTS, five chronological themes emerged: early pregnancy experiences, diagnostic experiences, making decisions, interventions and variable outcomes. These five themes can be contextualized within the framework of the TTTS experience as an “emotional rollercoaster”. In an account of her TTTS experience, Kennedy (2002) described TTTS as a “rollercoaster ride” [16]. She relayed the “devastating” impact of the diagnosis, the uncertainty of outcomes and experiencing extreme highs and lows including the heartbreak of the loss of one twin and elation as her other twin survived. Similarly, families in the current research depicted being pulled from the high of a twin pregnancy to the low of a TTTS pregnancy. Throughout the course of the disease experience, families were plagued by rapid changes in extreme emotional highs and lows. The unpredictable and variable course of the condition [4] coupled with the speed the condition can change likely contributed to the quickly changing emotional extremes.

### Early pregnancy experiences

At the beginning of the pregnancy, the majority of families recounted feelings of shock, excitement and fear at the discovery of twins. From this mostly emotional high, several families reported moving into feelings of anxiety with the sense that something was wrong. Almost one third of families described experiencing some type of indicator of TTTS that was missed by their physician. Another eight women indicated experiencing a type of mothers’ or bodily intuition that something was not right with the pregnancy. Four families never had time for intervention either due to a late or missed diagnosis and lost their babies. Not surprisingly, they expressed feelings of deep anger, sadness and regret. A potential sign of increased TTTS awareness within the medical community is that these four cases were limited to the early 2000’s (2003 and 2004).

However, TTTS is often misdiagnosed or diagnosis is delayed and these errors can have serious implications for mother and twins [5, 17]. Baud et al. (2014) report that between 2010 and 2012, 30 % of the patients who received laser treatment at a Canadian fetalscopy treatment center had been misdiagnosed by their prior healthcare providers [17]. Further, only 40 % of the expected 240 TTTS cases in Canada were referred to a fetalscopy center. As demonstrated by Nicholas (2014) in her study of patient-reported physician practices, lack of referral can have deadly consequences; patients who were not referred to a perinatologist after TTTS diagnosis were more likely to experience a double loss [5]. In the current study, two patients reported feeling ignored by their healthcare provider and needed to convince the provider there was a problem.

The need for patient self-advocacy to access appropriate TTTS diagnosis and treatment appears not uncommon. Several patients in the research conducted by Baud initiated the referral process to the treatment center as a result of their own information seeking [17]. Similarly, Nicholas found patients who felt the need to advocate for more care were more likely to have double survivors [5].

### Diagnostic experiences

For families with time for intervention, the experience of receiving a TTTS diagnosis was shocking, ending what had been typically portrayed as a joyful state. Sixteen percent of families were only given a name for the diagnosis or referral to a MFM specialist and were left to seek out treatment information on their own. An additional 16 % indicated they were informed by their providers that their babies would die. Almost half of all families described independently seeking out additional information to gain further insight into their ordeal. This may have helped families cope as seeking health information can reduce anxiety associated with health threats, help patients deal with unpredictability and is associated with emotional adjustment [18, 19]. Further, seeking health information can empower patients to participate with physicians during the decision-making process by providing patients with information about alternatives, assisting patients when choosing options, and reducing uncertainty related to decisions [20–22].

### Making decisions

Treatment options for TTTS include: termination of the pregnancy, selective reduction of one fetus, fetoscopic selective laser ablation surgery, amnio-reduction, septostomy, nutrition therapy, bed rest, and early delivery if the babies are viable [23–25]. While the gestational week in which a patient is diagnosed will affect the number of treatment options available, for instance, fetoscopic selective laser ablation surgery is often only performed between 16 and 26 weeks [26], the most common treatment choice is to undergo surgery. This is due primarily to the efficacy rate of the procedure, which is higher in both survival and decreased neurological impairment of survivors when compared to amnio-reduction [27].

In the present research, all parents with time for intervention described being provided options about how to proceed with the pregnancy. For some families the course of action was clear while for others decisions were less clear and second or third opinions were sought. One family recounted feeling pushed towards the decision to have surgery. Feeling a loss of control and the inability to question the decisions of a provider is an additional factor that can contribute to the already stressful nature of a high risk pregnancy [28].

Two families were left only with the option for selective reduction. One family relayed the devastation when faced with this choice and continued to search for options until they decided selective reduction was the only course of action to save one of the twins.

### Interventions

Although this number has been increasing, only a limited number of fetal treatment centers worldwide provide treatment for TTTS [29], and as seen in the current study, this necessitated travel away from home for nearly 42 % of the families in the current research. Financial hardships associated with unanticipated travel could result in additional stress for patients.

As indicated by the families in the current study, treatment provided opportunities for hope and simultaneously evoked fear and anxiety. Feelings of anxiety and fear are consistent with prior research demonstrating the emotional impact of fetal diagnosis and therapy [30]. Beck et al. found women undergoing fetal therapy were significantly more likely to experience mild to severe symptoms of depression compared to those undergoing diagnosis and assessment and 8 % required medical treatment for severe depression [30].

In the current study, families who had seemingly successful surgeries or series of amnio-reductions could still experience an abrupt change in prognosis with the reversal of the syndrome or sudden loss of one or both twins. Upon the reversal of the syndrome, one mother expressed feeling “defeated”, suggesting the experience of TTTS as a protracted battle against which coping resources may become drained. Analysis of journals among 11 women on hospitalized bedrest revealed high risk pregnancy was viewed by these mothers as a battle waged on a daily basis for the lives of their babies [31].

### Variable outcomes

Of the live births, the large majority spent time in the NICU. Approximately 61 % of those infants experienced no major complications. However, the remaining 38.7 % of infants suffered serious complications that could result in long-term special needs and increased stress for families [6]. Complications among survivors of TTTS are common, although information is primarily limited to children post-fetoscopic selective laser ablation surgery, and in fewer cases post-amnio-reduction treatment. Documentation of survivors with complications who did not undergo such treatments are not available. Among those children who were tracked and re-evaluated post-surgery, common complications include: cerebral palsy, psycho-motor developmental delay, borderline cognitive development, major neurodevelopmental impairment, motor development issues, blindness and deafness [32–34]. Given that fetoscopic selective laser ablation surgery is shown to produce more favorable outcomes, children who received no treatments may fair worse, however no known evidence exists.

Parents who lose one or more child as a result of TTTS are likely to be greatly impacted emotionally and psychologically by the experience. Bryan (2005) described how the parents, especially mothers, who have lost a twin during the perinatal period, will feel a great conflict of emotions [35]. In the current study, mothers who lost one twin directly to TTTS or afterwards in the NICU or at home, recounted the struggle to reconcile the profound sense of loss with the joy associated with the survivor. Further, because identical twins share nearly identical appearance, the sight of the surviving twin may be a constant reminder of the lost twin [35].

Parents who lose a twin during the prenatal period as a result of TTTS may not be given sufficient opportunity to grieve the loss of their child as all medical attention shifts to the survivor and parents are encouraged to do the same. In the case of in-utero death during TTTS pregnancy, it is logical that specialists and physicians quickly shift their focus to the surviving twin. However, families need the chance to cope with the loss. Further, because the lost twin may have passed away weeks or months prior, during delivery providers may no longer recognize the pregnancy as a twin gestation and fail to consider the emotional trauma of giving birth to the lost twin. Additionally, while limited research examines the emotional impact associated with the loss of both TTTS twins, it is anticipated that parents experiencing double loss may be at risk for long-standing bereavement, feelings of isolation, higher risk for clinical depression and anxiety as well as the perception of a lack of social and medical support. [36–40] Consequently, during TTTS pregnancies providers should be sensitive to the family’s loss and prepare themselves to respect family bereavement.

### Limitations

Because the present research used existing, publicly accessible TTTS stories, the stories may lack generalizability and possibly overrepresent families who experienced more severe cases or were more emotionally affected by the TTTS experience. However, several factors may have contributed to the underrepresentation of negative outcomes. First, outcomes shared by families include survival rates similar to and, in most instances, higher than rates reported by previous research [4]. Second, work by Nicholas demonstrated disparities in TTTS diagnosis and poor survival outcomes among minorities, individuals of lower socioeconomic status and those with lower educational attainment. Because minorities and individuals in rural areas are less likely to have home computers and access to the internet [41], individuals experiencing health disparities and negative TTTS outcomes may have been less likely to share their stories online. Third, not all families may be aware that they lost a pregnancy as a result of TTTS due to problems with misdiagnosis and lack of access to appropriate care [17]. Together these factors suggest that the current research may have underrepresented the proportion of families who experience a single or double loss.

Further, while bias due to interaction between researcher and participant was absent since this research examined spontaneously posted online stories [42], the first author’s personal experiences as a mother who experienced a TTTS pregnancy may be an inherent source of subjectivity. However, recognizing this possibility, the first author took steps to mitigate this influence, including using a second analyst and involving another TTTS researcher in the project [43].

## Conclusion

The experience of an “emotional roller coaster” ride through the high risk pregnancy is not unique to the TTTS disorder. Prenatal diagnostic screening for genetic or fetal anomalies [44], pregnancies at high risk for pre-term birth [45], hospitalization during hypertension [46], and pregnancy and parenting after a stillborn [47] are just a few examples from the literature which have been similarly characterized.

However, there are several important aspects about the common TTTS disease experience, highlighted in the current study, that merit attention from researchers and healthcare providers alike. TTTS is a highly unpredictable, quickly changing disorder [4] and, as illustrated in the current study, outcomes can change in the course of a single day. This research also revealed a clear pressure to make quick decisions about the pregnancy in a “race against time”. In addition, families often must travel away from home to seek treatment with little notice. It is plausible that this unexpected travel and treatment could incur financial hardships. While coping mechanisms and their efficacy may differ among the women and families who experience TTTS, it is feasible that the disorder would be associated with elevated stress levels and potential for psychological impacts. Further, perinatal loss as a result of TTTS may lead to profound psychological distress including risk for major depression [48] and post-traumatic-stress disorder (PTSD) [49]. For families with TTTS survivors who enter the NICU after birth, additional stressors and emotional challenges must be met, in particular among mothers with more premature infants and longer NICU stays [50]. Among parents who have special needs children as a result of TTTS, these families may experience higher than typical stress levels and emotional challenges [6].

The current research provides the foundation and a theoretical model to support the further exploration of the TTTS experience, including potential emotional and psychosocial impacts. Future research should examine the levels of stress associated with this syndrome and the various outcomes as well as explore individual differences in coping mechanisms and subsequent psychological burden. Future research should also explore the ways healthcare providers can best meet the specific emotional needs at each point and for all outcomes of the TTTS experience. Counseling, referral to grief support teams, support groups and psychiatric services can be beneficial to families after perinatal loss [49], during NICU stays [51], among families with special needs children [52] and during high risk pregnancy [53] and may be useful to all families during each phase of the TTTS journey.

## Abbreviations

TTTS, Twin-to-twin transfusion syndrome; MCDA, Monochorionic diamniotic; MFM Maternal fetal medicine; NICU Neonatal intensive care unit.

## Footnotes

1 Names have been changed throughout the text.
